# The Prognostic Potential of PD-L1, PD-1, CD3, CD4, and CD8 Expression in Patients with Head and Neck Cancers Depending on HPV16 Infection

**DOI:** 10.3390/cancers18111771

**Published:** 2026-05-28

**Authors:** Anna Mucha-Małecka, Beata Biesaga, Natalia Amrogowicz, Aleksandra Grela-Wojewoda, Mirosława Püsküllüoğlu, Marcin Przewoźnik, Elżbieta Pluta, Anna Patla, Krzysztof Roszkowski, Krzysztof Małecki

**Affiliations:** 1 Department of Radiotherapy, Maria Sklodowska-Curie National Research Institute of Oncology, Krakow Branch, Garncarska Street 11, 31-115 Cracow, Poland; elzbieta.pluta@krakow.nio.gov.pl (E.P.); anna.patla@krakow.nio.gov.pl (A.P.); 2 Department of Tumor Pathology, Maria Sklodowska-Curie National Research Institute of Oncology, Krakow Branch, Garncarska Street 11, 31-115 Cracow, Poland; beata.biesaga@krakow.nio.gov.pl (B.B.); marcin.przewoznik@krakow.nio.gov.pl (M.P.); 3 First Radiation and Clinical Oncology Department, Maria Sklodowska-Curie National Research Institute of Oncology, 44-102 Gliwice, Poland; natalia.amrogowicz@gliwice.nio.gov.pl; 4 Department of Clinical Oncology, Maria Sklodowska-Curie National Research Institute of Oncology, Krakow Branch, Garncarska Street 11, 31-115 Cracow, Poland; aleksandra.grela@krakow.nio.gov.pl (A.G.-W.); miroslawa.puskulluoglu@krakow.nio.gov.pl (M.P.); 5 Department of Oncology, Collegium Medicum, Nicolaus Copernicus University, 85-821 Bydgoszcz, Poland; roszkowskik@cm.umk.pl; 6 Department of Radiotherapy for Children and Adults, University Children’s Hospital of Cracow, Wielicka 265, 30-663 Cracow, Poland; kmalecki@usdk.pl

**Keywords:** head and neck cancers, HPV16, prognostic biomarkers, immune checkpoints

## Abstract

**Objective:** The aim of this study was to evaluate the expression of PD-L1, PD-1, CD3, CD4, and CD8 in tumor tissues of patients with head and neck squamous cell carcinoma from southern Poland, and to assess their prognostic value in relation to disease-free survival (DFS), taking into account HPV16 status and other clinical, pathological, and demographic factors. **Material/Methods:** This study included 155 unselected patients with head and neck squamous cell carcinoma (HNSCC) from the southern Poland region, who underwent diagnostic evaluation and surgical treatment. Formalin-fixed, paraffin-embedded (FFPE) tissue blocks were obtained from these centers. The patients were treated at the Maria Skłodowska-Curie National Research Institute of Oncology, Kraków Branch, between 1991 and 2014, with treatment approaches including induction therapy (preoperative), adjuvant therapy (postoperative), or definitive chemoradiotherapy with cisplatin. Protein expression was assessed using immunohistochemistry and quantified (TPS, CPS, H-score). Relationships between expression levels and epidemiological, clinical, and histopathological features were analyzed. **Results:** The results show that PD-L1 overexpression was associated with worse DFS, whereas overexpression of PD-1, CD3, CD4, and CD8 correlated with improved DFS. These associations were statistically significant in the HPV16-negative subgroup, while no such correlations were found in HPV16-positive patients. In multivariate analysis, independent prognostic factors associated with improved DFS included HPV16 infection, absence of PD-L1 overexpression, overexpression of CD4 and CD8, and combined chemoradiotherapy with cisplatin. **Conclusions:** These findings confirm the prognostic relevance of PD-L1, PD-1, and T-cell markers in HNSCC, particularly in HPV16-negative patients, and support further research into the use of these biomarkers in personalized treatment strategies.

## Simple Summary

Head and neck squamous cell carcinoma is a common cancer with highly variable clinical outcomes, highlighting the need for reliable biomarkers that may help predict prognosis and guide treatment decisions. In this study, we investigated the expression of immune-related proteins, including programmed death-ligand 1, programmed cell death protein 1, and T-cell markers, in tumor tissues from patients in southern Poland. We also examined whether these markers were associated with human papillomavirus type 16 infection and patient outcomes. Our results show that high programmed death-ligand 1 expression was linked to poorer disease-free survival, whereas increased infiltration of immune cells was associated with better prognosis, particularly in patients without human papillomavirus infection. These findings improve understanding of the tumor immune microenvironment in head and neck cancer and may support the future development of personalized immunotherapy strategies.

## 1. Introduction

Head and neck squamous cell carcinoma (HNSCC) is the sixth most commonly diagnosed malignant neoplasm worldwide and represents a major public health concern, particularly due to its marked biological heterogeneity and variability in treatment response. Despite advances in surgical management, radiotherapy, and systemic therapies, the prognosis of patients with HNSCC remains unsatisfactory, especially in advanced stages of the disease. Five-year survival rates remain low, and the high risk of local recurrence and distant metastasis continues to pose a significant clinical challenge [1].

In recent years, increasing attention has been focused on the role of the tumor microenvironment and the interactions between immune cells and tumor cells in the pathogenesis and progression of HNSCC. Particular emphasis has been placed on the PD-1/PD-L1 signaling pathway, which mediates tumor-associated immunosuppression and enables immune escape. At the same time, markers such as CD3, CD4, and CD8 reflect the presence and activity of T lymphocytes and may serve as potential indicators of the host immune response [2,3].

These biomarkers are gaining importance not only as prognostic indicators but also as predictive markers, particularly in the context of immunotherapy [2].

In addition to immune checkpoint molecules, markers of lymphocytic infiltration such as CD3, CD4, and CD8 are increasingly recognized as important prognostic and potentially predictive factors. These markers reflect both the activity and composition of the local immune response within the tumor microenvironment and may be associated with improved clinical outcomes in several solid malignancies, including head and neck squamous cell carcinoma (HNSCC) [4]. Furthermore, in oropharyngeal squamous cell carcinoma, tumor biology and immune landscape are strongly influenced by the presence of transcriptionally active Human papillomavirus (HPV), most commonly HPV16. HPV-positive tumors are characterized by distinct molecular features, increased immune infiltration, and a more favorable response to treatment compared with HPV-negative disease. Consequently, HPV status has emerged as a critical determinant of prognosis and survival outcomes and is now considered an essential stratification factor in contemporary clinical and translational oncological research [5,6,7,8,9].

Therefore, the aim of this study was to evaluate the expression of PD-L1, PD-1, CD3, CD4, and CD8 in tumor samples obtained from 155 patients with HNSCC treated in southern Poland and to analyze their association with patients’ clinical, pathological, and epidemiological characteristics, including the presence of HPV16 infection [10]. Due to insufficient quality of the extracted DNA in a subset of cases, reliable analysis was possible in 151 patients. In the remaining 4 cases, HPV16 assessment could not be performed. Additionally, the potential prognostic significance of these markers was assessed in exploratory analysis in the context of five-year disease-free survival (DFS) using both univariate and multivariate analyses.

## 2. Materials and Methods

### 2.1. Qualification of Patients for the Study

The study included 155 unselected patients with head and neck squamous cell carcinoma (HNSCC) from the southern Poland region (Małopolska), who underwent diagnostic evaluation and surgical treatment in otolaryngology departments of various hospitals in Małopolska. These primarily included the 5th Military Clinical Hospital with Polyclinic in Kraków, Ludwik Rydygier Specialist Hospital in Kraków, and Jagiellonian University Medical College in Kraków. Formalin-fixed, paraffin-embedded (FFPE) tissue blocks were obtained from these centers. The patients were treated at the Maria Skłodowska-Curie National Research Institute of Oncology, Kraków Branch, between 1991 and 2014, with treatment approaches including induction therapy (preoperative), adjuvant therapy (postoperative), or definitive chemoradiotherapy with cisplatin.

Inclusion criteria were as follows: (1) primary tumor localization in the head and neck region, (2) squamous cell carcinoma histology, and (3) no prior cancer treatment. Patients with distant metastases at diagnosis were excluded from the analysis.

For all participants, data on smoking (expressed as the number of cigarettes per day multiplied by years of smoking) and alcohol consumption (classified as low or high) were collected, along with demographic (age, sex, Karnofsky performance score), clinical (tumor localization, clinical stage, lymph node metastasis, treatment), and histopathological data (tumor grade and keratinization). Additionally, in this patient cohort, transcriptionally active HPV16 infection was assessed based on prior analyses [9]. However, due to insufficient quality of the extracted DNA in a subset of cases, reliable analysis was possible in 151 patients. In the remaining 4 cases, HPV16 assessment could not be performed. These analyses utilized DNA isolated from archival FFPE tumor tissues and included nested PCR, qPCR (HPV genotyping), and immunohistochemical evaluation of p16 protein expression.

The study was conducted in accordance with the approval of the Bioethics Committee at the District Medical Chamber in Kraków (approval no. 5/KBL/OIL/2017). All participants provided written informed consent for the use of their anonymized data for research purposes.

### 2.2. Material

The study was conducted based on archival fragments of tumor tissue, tumor tissue obtained during surgery or biopsy and subsequently fixed in paraffin and embedded in paraffin. For each patient, a series of such blocks was collected. For every patient, using standard histopathological slides stained with hematoxylin and eosin, a pathologist confirmed the presence of squamous cell carcinoma, assessed the histopathological malignancy grade, the degree of tumor keratinization, and identified a block containing tissue with at least 50% tumor composition in the section for further molecular and immunohistochemical (IHC) analyses.

### 2.3. Immunohistochemistry Staining

Sections underwent deparaffinization, rehydration, and antigen unmasking by heating in citrate buffer (10 mM sodium citrate, 0.05% Tween 20, pH 6.0) at 96 °C for 50 min. Endogenous peroxidases were quenched by incubation in 0.3% hydrogen peroxide at 37 °C for 30 min. Following this, slides were incubated for 90 min at 37 °C with diluted primary antibodies overnight at 4 °C (monoclonal, mouse, anti-human PD-L1, clone 22C3, dilution 1:50, DAKO; monoclonal, rabbit, ani-human PD-1, Invitrogen, Thermo Fisher Scientific, Waltham, MA, USA) dilution 1:200; polyclonal, rabbit anti-human CD3, Dako Denmark A/S, Glostrup|Denmark, dilution 1:100; monoclonal, mouse, anti-human CD4, clone 4B12, Dako Denmark A/S, Glostrup, Denmark, dilution 1:50; monoclonal, mouse, anti-human CD8, clone C8/144B, Dako Denmark A/S, Glostrup|Denmark, dilution 1:100). The reaction was visualized using the BrightVision system (Immunologic, Duiven, The Netherlands) and 0.01% 3,3′-diaminobenzidine tetrahydrochloride (Vector Laboratories, Burlingame, CA, USA). Slides were counterstained with Mayer’s hematoxylin. For each staining run, both positive and negative controls were included. Positive controls consisted of a tonsillar carcinoma specimen demonstrating a consistently strong immunohistochemical reaction, selected in accordance with the manufacturers’ recommendations for all primary antibodies used. Negative controls were processed in parallel for each staining series by omission of the primary antibody incubation step.

### 2.4. Immunohistochemistry Staining Assessment

**Figure 1:**
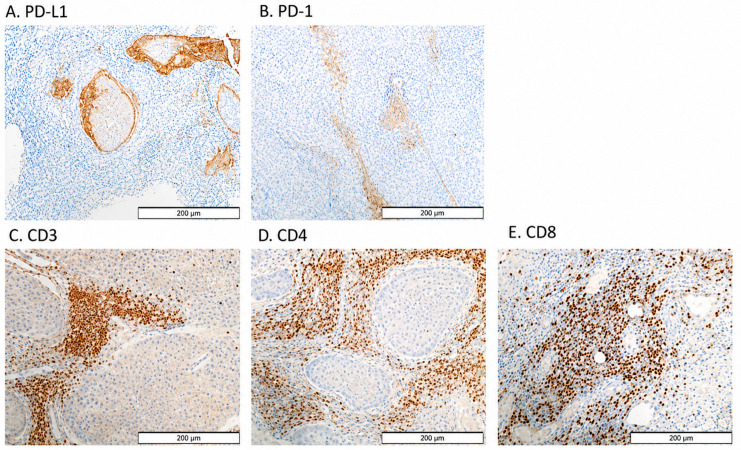
Immunohistochemical analysis of immune checkpoint markers and T-cell subsets in head and neck tumors. (**A**) PD-1 expression. (**B**) PD-L1 expression. (**C**) CD3^+^ T lymphocytes. (**D**) CD4^+^ T helper cells. (**E**) CD8^+^ cytotoxic T cells. Brown staining indicates positive immunoreactivity. Representative images shown at ×200 magnification.

For IHC staining related to PD-L1 expression, microscopic analysis focused on evaluating two parameters: TPS (Tumor Proportion Score) and CPS (Combined Positive Score) according to [11,12] (Figure 1A). TPS measures the percentage of viable tumor cells in a sample that shows PD-L1 staining on their membranes. It does not include immune cells or other cell types. CPS, also applied in the case of membranous PD-1 expression, accounts for expression in both tumor cells and immune cells (including lymphocytes and macrophages) (Figure 1B). It reflects the overall tumor microenvironment’s contribution to immune evasion. For other immunohistochemical stains (CD3, CD4, and CD8), the analysis assessed membranous staining intensity and the percentage of stained tumor cells (Figure 1C–E). Interobserver reliability was assessed by two independent observers (B.B. and M.P.) who evaluated the immunohistochemical staining results. The agreement between the observers was high, and any discrepancies were resolved by joint review and consensus. Staining intensity was evaluated using a 3-point scale: 1—no staining or weak staining, 2—moderate staining, 3—strong staining. The stained samples were evaluated by two independent researchers. For all proteins, the H-score (HS) value was calculated using the following formula: HS = (1 × % of cells stained with intensity) + (2 × % of cells stained with intensity) + (3 × % of cells stained with intensity. This method was chosen due to its ability to compare the obtained results with those of other authors and its unique consideration of tumor heterogeneity. The expression of each examined protein was classified as “overexpression” for tissues with an HS higher than the cut-off value or “no overexpression” for tissues with an HS equal to or lower than the cut-off value (including tissues with no expression).

### 2.5. Statistical Analysis

To determine the mean, standard error of the mean (SE), and median for continuous variables, descriptive statistics were used. Relationships between categorical variables were analyzed using Pearson’s chi-square test. Cut-off points were calculated using the minimal *p*-value method. Prior to cut-off determination, we performed a comprehensive descriptive statistical analysis for each marker, including mean, median, minimum and maximum values, as well as the 25th and 75th percentiles, in order to fully characterize the distribution of expression levels within the study cohort.

Prognostic potential was analyzed based on 5-year DFS (disease-free survival, defined as the time from the end of treatment to the first documented disease progression, i.e., treatment failure, local recurrence, or distant metastasis occurring within 5 years after treatment completion). DFS probabilities were calculated using the Kaplan-Meier method and compared using the log-rank test.

To identify independent prognostic factors in the studied patient group, univariate and multivariate analyses were performed based on the Cox proportional hazards model. All parameters that had a statistically significant impact on patient survival in the univariate analysis were subsequently included in the multivariate analysis.

Calculations were performed using the Statistica v.13.3 software. Differences were considered statistically significant at *p* < 0.05.

## 3. Results

### 3.1. Patient Characteristics

A group of 155 patients with squamous cell carcinoma of the head and neck was included in the study, comprising 25 individuals with oral cancer (16.1%), 66 with oropharyngeal cancer (42.6%), 6 with hypopharyngeal cancer (3.9%), and 58 with laryngeal cancer (37.4%) (Table 1). The mean age of the patients was 56.9 years ± 9.4 (SE), with a median age of 59 years (range: 24–78 years). The majority of patients in the study group had clinical stage T3 (50.3%) and N2 (54.2%) of disease. HPV16 status was successfully assessed in 151 patients; in 4 cases, analysis could not be performed due to insufficient quality of the extracted DNA from archived material. The combined evaluation of viral DNA presence and p16 overexpression enabled identification of a subgroup of 28 patients (18.5%) with transcriptionally active HPV16 infection [9].

Among the 155 patients included in the study, 88 individuals (56.8%) received postoperative radiotherapy (RT) or RT alone. The total RT dose ranged from 20.0 to 66.0 Gy, with a mean of 59.1 Gy ± 2.6, delivered in 5–40 fractions, with fraction doses ranging from 1.8 to 4.0 Gy. Concurrent chemoradiotherapy with cisplatin (CRT-CisPt) was administered to 45 patients (29.0%) as either definitive treatment or adjuvant therapy following surgery. In this subgroup, RT doses ranged from 28 to 70 Gy (mean: 64.1 Gy ± 1.6), delivered in 14–35 fractions with fraction sizes of 2.0–2.2 Gy. Cisplatin (CisPt) was delivered during RT according to two dosing regimens: (1) 100 mg/m^2^ every three weeks for two to three cycles or (2) 40 mg/m^2^ weekly for three to six cycles, based on the patient’s clinical condition and the severity of early treatment-related toxicities. Additionally, 22 patients (14.2%) underwent induction chemotherapy with cisplatin, 5-fluorouracil, and taxanes, followed by RT. The RT dose in this group ranged from 28 to 70 Gy, with a mean of 64.7 Gy ± 2.7, delivered in fraction doses of 2.0 Gy over 14–35 fractions.

Five years after the completion of treatment, 68 patients (43.9%) were alive, 50 (32.3%) died due to cancer, and 37 (23.8%) died from other causes, primarily cardiovascular diseases. Cancer regression was observed in 61 patients (39.4%), while disease progression occurred in 57 patients (36.8%). Among these, treatment failure was noted in 34 cases, locoregional recurrence in 16 cases, and distant metastases in 7 patients. Disease progression was observed within 0 to 91 months after treatment completion, with a mean duration of 19.8 months ± 4.1 and a median of 12.5 months.

### 3.2. PD-L1, PD-1, CD3, CD4 and CD8 Expression in the Group of 155 Patients Included in the Study

In the group of 155 patients included in the study, positive staining for PD-L1 expression in tumor cells and immune cells was observed in subgroups of 21 (13.5%) and 42 (27.1%) patients, respectively. The mean and median values of positive staining cells were 1.8 and 0.0 for PD-L1 TPS and 5.5 and 0.0 for PD-L1 CPS (Table 1). For both PD-L1 TPS and PD-L1 CPS, the optimal cutoff point (most effectively differentiating survival curves) was determined to be 5.0. These cutoff points identified the same subgroup of 30 patients (19.4%) with PD-L1 overexpression based on both TPS and CPS.

In the group of 155 patients, positive staining for PD-1 was observed in all analyzed tumors. The mean and median values for PD-1 CPS were 11.7 and 11.0, respectively. Using the minimal *p*-value method, an optimal cutoff point of 11.0 was determined, allowing identification of a subgroup of 99 patients (63.9%) with PD-1 overexpression.

For CD3, CD4, and CD8, positive IHC staining was found in the subgroups of 148 (95.4%, mean and median HS = 67.7 and 64.0), 133 (85.8%, mean and median HS = 54.2 and 55.0), and 150 patients (96.8%, mean and median HS = 78.1 and 75.0), respectively. The cutoff points for CD3, CD4, and CD8 determined by the minimal *p*-value method were 65.0, 105, and 140, respectively. Based on these cutoff points, subgroups of patients with CD3 (*n* = 79, 51.0%), CD4 (*n* = 32, 20.6%), and CD8 (*n* = 50, 32.3%) overexpression were identified in the analyzed cohort.

### 3.3. Analysis of the Relationships Between Protein Expression and Epidemiological, Clinical, and Histopathological Features

An analysis of the relationships between protein expression and epidemiological, clinical, and histopathological features was conducted. PD-L1 expression was significantly associated with smoking status, tumor grade, PD-1 and CD3 expression (Table 1). Overexpression of PD-L1 was also significantly more common in heavy smokers, as well as in tumors with higher grades and absent PD-1 and CD3 overexpression. No significant correlations between PD-1. Significant interrelation between CD3, CD4, and CD8 overexpression was detected: the absence of CD4 overexpression was significantly associated with the absence of CD8 overexpression, while CD3 overexpression was correlated with CD8 overexpression. HPV16 infection significantly influenced the occurrence of CD3, CD4, and CD8 overexpression, with infection being significantly associated with CD3 and CD4 overexpression but not with CD8 overexpression. Significant differences in CD4 expression were also observed depending on Karnofsky performance status and tumor location. Among the patients analyzed, the most significant relationships were observed between epidemiological, clinical, and histopathological features and CD8 levels. Overexpression of CD8 was significantly more frequent in older patients, females, and tumors at T2 and T3 stages of clinical advancement. Statistically significant differences in the expression of all analyzed proteins were observed depending on treatment outcomes, with protein overexpression being associated with better outcomes.

### 3.4. Survival Analysis and Independent Prognostic Factors

**Figure 2:**
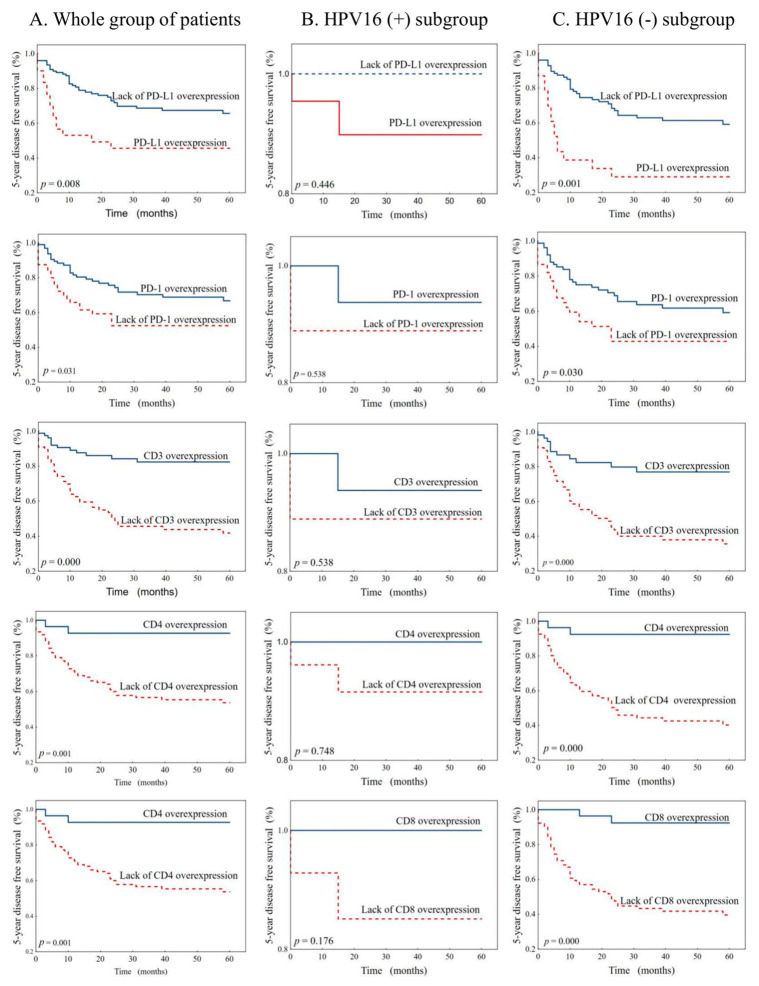
Association between immunohistochemical expression of PD-L1, PD-1, CD3, CD4, and CD8 and disease-free survival in 155 Patients with head and neck malignancies: total population (**A**), HPV16(+) (**B**), and HPV16(-) (**C**).

In the cohort of 155 patients, the 5-year DFS rate was 42.9%. Univariate analysis revealed that the absence of PD-L1 overexpression and the presence of PD-1, CD3, CD4, and CD8 overexpression were significantly correlated with higher DFS (Table 2, Figure 2A). Additionally, older patients, females, and individuals with lower levels of smoking and alcohol consumption also exhibited significantly higher DFS. Clinical factors positively influencing DFS included lower T-stage, the presence of HPV16 infection, and concurrent chemoradiotherapy with cisplatin (CRT with CisPt).

Separate survival analyses were conducted in patients with HPV16-positive (Figure 2B) and HPV16-negative tumors (Figure 2C). In the HPV16-negative subgroup, the absence of PD-L1 overexpression and the presence of PD-1, CD3, CD4, and CD8 overexpression were significantly associated with higher 5-year DFS. In this subgroup, higher T-stage also had a significant positive impact on DFS. In the HPV16-positive subgroup, none of the analyzed proteins, as well as epidemiological, clinical, or histopathological features significantly influenced DFS.

In the cohort of 155 patients and in the HPV16-negative subgroup, a multivariate Cox analysis was performed. This analysis included factors that significantly influenced DFS in the univariate analysis. For the entire cohort, these factors were: age, gender, smoking and alcohol consumption levels, HPV16 infection status, and expression levels of PD-L1, PD-1, CD3, CD4, and CD8. Among these, independent prognostic factors significantly improving DFS included HPV16 infection, absence of PD-L1 overexpression, CD4 and CD8 overexpression, and concurrent CRT with CisPt (Table 3). In the HPV16-negative subgroup, the multivariate Cox analysis included T-stage and the expression levels of PD-L1, PD-1, CD3, CD4, and CD8. Among these, independent prognostic factors significantly affecting DFS were the absence of PD-L1 overexpression and CD4 and CD8 overexpression.

## 4. Discussion

The study analyzed the expression of immune checkpoint molecules PD-L1 and PD-1, as well as key tumor-infiltrating lymphocyte (TIL) markers, namely CD3, CD4, and CD8, in a cohort of 155 patients with HNSCC. The results confirm that the tumor immune microenvironment is a critical determinant of disease progression and prognosis, particularly in HPV16-negative tumors. These observations are consistent with the concept of “immune contexture” as a key prognostic factor in solid tumors [13].

### 4.1. Prognostic Significance of PD-L1 Expression

In our cohort, PD-L1 overexpression (CPS ≥ 20) was observed in approximately 20% of tumors, a similar frequency to that reported by Mishra et al. [14]. In other HNSCC studies, high PD-L1 expression was reported in 25.6% to 43.7% of cases [15,16,17]. These findings are consistent with published literature: a meta-analysis demonstrated a wide range of PD-L1 positivity in HNSCC (average ~42% across different cut-offs, including lower frequencies with more stringent criteria) [18]. Other analyses indicate that the frequency of PD-L1 expression in HNSCC can be high, but depends on the applied evaluation criteria, which explains discrepancies between studies [19]. These results confirm that a substantial subset of HNSCC tumors exhibits high PD-L1 expression across different populations and methodological conditions.

Our study revealed associations between PD-L1 overexpression, heavy tobacco exposure, and higher histological grade. These findings are consistent with previous reports, such as Li et al. [20], who showed that tobacco smoking is linked to increased PD-L1 expression, suggesting that carcinogens in tobacco smoke may modulate immune checkpoint pathway activation. Similarly, high PD-L1 expression and enhanced immune checkpoint signaling are frequently observed in HNSCC, supporting the notion that tobacco exposure can shape the tumor immune microenvironment [21]. Tobacco use was found to affect immune cell infiltration and immune signaling pathways in HNSCC, indicating smoking-related modulation of immune checkpoint pathways [22].

In the analyzed material, the absence of PD-L1 overexpression was independently associated with better 5-year disease-free survival (DFS), suggesting that activation of the PD-1/PD-L1 axis may play a significant role in disease progression in a subset of patients with HNSCC. Although the prognostic value of PD-L1 in HNSCC remains a subject of debate, numerous studies have demonstrated worse survival outcomes in patients with PD-L1-positive tumors, indicating an association between PD-L1 expression and a more aggressive tumor phenotype [18,23,24,25,26].

However, discrepancies among literature reports are largely a consequence of substantial methodological heterogeneity, including differences in the antibody clones used, expression assessment systems (TPS vs. CPS), adopted cut-off values, and intratumoral variability in PD-L1 expression [27]. Therefore, PD-L1 should not be interpreted as an isolated prognostic biomarker, but rather as a component of a complex network of interactions between tumor cells and the immunological tumor microenvironment.

### 4.2. PD-1 Expression and Activation of the Immune Response

PD-1 expression was detected in all analyzed tumors, indicating the presence of an activated immune infiltrate and confirming that immunoregulation within the tumor microenvironment is preserved in HNSCC. This finding is consistent with previous observations in HNSCC and other solid tumors, where high PD-1 expression has often been shown to correlate with the presence of functional T lymphocytes, which may reflect an active antitumor response rather than merely a state of T-cell exhaustion [28,29].

In univariate analysis, higher PD-1 expression was associated with improved disease-free survival (DFS), suggesting that PD-1 may reflect an ongoing antitumor response rather than irreversible T-cell exhaustion. However, in multivariate analysis, PD-1 did not retain independent prognostic significance, indicating that its impact on survival may be secondary to overall lymphocyte density and the coordination of the immune response within the tumor microenvironment.

These results highlight the importance of assessing coordinated immune infiltration rather than isolated expression of individual immune checkpoints when predicting the clinical course of HNSCC. The coexistence of high PD-1 expression with the presence of TIL markers (CD3, CD4, CD8) suggests a functional immune response that may support the effectiveness of immunomodulatory therapies, including PD-1/PD-L1 inhibitors [28,29,30,31].

### 4.3. Tumor-Infiltrating Lymphocytes—Prognostic Significance

The strong prognostic value of TIL markers observed in the present study is consistent with extensive literature indicating a beneficial impact of immune infiltration in HNSCC [32,33]. Overexpression of both CD4 and CD8 constituted independent predictive factors for improved disease-free survival (DFS), highlighting the complementary roles of helper and cytotoxic T lymphocytes in an effective antitumor response. Moreover, the observed correlation among CD3, CD4, and CD8 expression supports the concept of coordinated immune infiltration, reflecting a functional antitumor response rather than the isolated presence of individual lymphocyte populations [32,33].

CD8 overexpression showed a particularly strong association with favorable clinical outcomes, which is consistent with previous studies identifying CD8^+^ lymphocytes as key effectors of tumor control and important predictors of treatment response [34,35]. These findings underscore the role of cytotoxic lymphocytes in the elimination of cancer cells and highlight their potential significance in predicting therapeutic efficacy, including immunotherapy.

### 4.4. Impact of HPV16 Status and Clinical Implications

HPV16-positive status has been confirmed as a strong favourable prognostic factor, consistent with numerous reports demonstrating significantly improved treatment outcomes in patients with HPV-related oropharyngeal squamous cell carcinoma [5]. In the analyzed cohort, HPV16-positive tumors were characterized by higher expression of T-cell markers, including CD3 and CD4, corresponding to an inflammatory immune phenotype typical of virally driven malignancies and reflecting an active immune response directed against viral antigens [36,37].

Despite this pronounced immune activation, none of the analyzed immunological markers showed a significant impact on disease-free survival (DFS) within the HPV16-positive subgroup. Similar observations have been reported by other authors, who indicated that, in HPV-positive tumors, additional prognostic stratification based on lymphocytic infiltration provides limited incremental value [38]. This phenomenon may reflect a so-called ceiling effect, whereby the inherently favorable tumor biology and high treatment sensitivity of HPV-associated cancers constrain the additional prognostic contribution of individual immunological parameters.

In contrast, HPV16-negative tumors are characterized by greater biological heterogeneity, poorer prognosis, and a more diverse immune microenvironment. In this subgroup, immunological parameters such as the degree of T-cell infiltration—including CD4^+^ and CD8^+^ T-cell populations—as well as PD-L1 expression, may provide additional prognostic and predictive information by identifying patient subgroups with distinct clinical courses [36,37]. These findings underscore the importance of biomarker analyses performed in the context of HPV status.

From a clinical perspective, the present results suggest the integration of immunological profiling into prognostic stratification, particularly in patients with HPV16-negative head and neck squamous cell carcinoma (HNSCC). PD-L1 expression currently represents an important predictive biomarker for response to immune checkpoint inhibitor therapy, which has demonstrated clinical efficacy in the treatment of recurrent and metastatic head and neck squamous cell carcinoma [2]. Moreover, the favorable impact of CD4^+^ and CD8^+^ T-cell infiltration observed in selected cohorts further suggests the rationale for therapeutic strategies aimed at modulating and enhancing antitumor immune responses, especially in HPV-negative tumors, in which the effectiveness of standard treatment modalities remains limited.

However, the present study has several limitations, including temporal heterogeneity of the cohort, variability in clinical characteristics, and a relatively small number of HPV16-positive cases. These factors should be taken into consideration when interpreting the results.

With respect to treatment heterogeneity, it should be noted that in our cohort, no significant associations were observed between treatment modality and the expression levels of the analyzed biomarkers. Nevertheless, existing literature suggests a potential relationship between treatment type and immune-related biomarker expression. For instance, Guerini Rocco et al. [39] reported that in patients treated with adjuvant chemoradiotherapy following surgery, higher PD-L1 expression may be associated with improved overall survival compared with low PD-L1 expression. However, to the best of our knowledge, no studies have simultaneously evaluated PD-L1, PD-1, CD3, CD4, and CD8 expression in relation to treatment modality in HNSCC.

Importantly, our study is, to our knowledge, the first to assess the prognostic relevance of these immune markers in relation to HPV status within a heterogeneous clinical cohort. Therefore, the present analysis should be regarded as exploratory in nature, generating hypotheses that require validation in larger, independent, and more homogeneous patient populations.

Considering heterogeneity according to clinical characteristics, it should be noticed that HNSCC comprises a biologically and clinically heterogeneous group of tumors arising from distinct anatomical subsites, which differ with respect to etiological factors, prognostic behavior, the relevance of HPV infection, and therapeutic approaches. This heterogeneity may potentially affect the interpretability of pooled analyses. HNSCC, despite subsite-specific differences, represents a unified entity with shared molecular and immunological characteristics, particularly with regard to tumor-immune microenvironment interactions, which were the primary focus of the present study. This is supported by multiple published studies demonstrating that key immune biomarkers, including PD-L1 expression, are present across different head and neck subsites, albeit with variable frequency. At the same time, the rationale for combining all subsites into a single cohort was driven by both biological and methodological considerations.

Importantly, the present study was designed as an exploratory analysis. Its primary aim was to identify potential immunological and prognostic patterns across the entire HNSCC spectrum rather than to perform definitive subsite-specific stratification. Accordingly, the combined analysis was considered appropriate for hypothesis-generating purposes, with the intention of providing a broader overview of immune marker behavior in routine clinical material.

The HPV16-positive subgroup in our study was relatively small (n = 28), which limits the statistical power of subgroup analyses, particularly in survival and multivariable models. Therefore, all conclusions regarding this subgroup should be interpreted with appropriate caution and considered exploratory in nature. However, it should be noticed that despite the limited sample size, the observed finding that HPV16-positive status is associated with a favorable prognosis is fully consistent with a large body of published literature demonstrating significantly improved treatment outcomes in patients with HPV-related oropharyngeal squamous cell carcinoma [5]. This strong and well-established prognostic effect supports the biological validity of the observed subgroup characteristics in our cohort.

Finally, attention should be paid to the minimal *p*-value method used to determine the cut-off points for all biomarkers. This method is related to increased risk of overfitting and reduced reproducibility of derived cut-off values. However, prior to cut-off determination, we performed a comprehensive descriptive statistical analysis for each marker, including mean, median, minimum and maximum values, as well as the 25th and 75th percentiles, in order to fully characterize the distribution of expression levels within the study cohort.

Despite the acknowledged limitations related to intratumoral heterogeneity assessment, temporal variability of the cohort, and its retrospective design, the present study has several important strengths.

First, the analysis was based on a relatively large, well-characterized cohort of 155 patients with head and neck squamous cell carcinoma, with comprehensive clinicopathological and follow-up data collected over a long observation period. Importantly, all cases were histopathologically verified, and tissue selection was standardized through centralized pathological review, ensuring a high level of diagnostic consistency.

Second, the study provides a broad, integrative evaluation of multiple components of the tumor immune microenvironment, including PD-L1/PD-1 axis markers as well as key T-cell populations (CD3, CD4, CD8), analyzed in parallel within the same cohort. This allows for a more comprehensive assessment of immune contexture compared with studies focusing on single biomarkers.

Third, the inclusion of HPV16 status in conjunction with immune markers provides additional biological stratification and enables the exploration of interactions between viral status and tumor immune infiltration, which remains insufficiently characterized in HNSCC.

Finally, the study reflects real-world clinical heterogeneity in terms of tumor localization and treatment modalities, which enhances the external validity and translational relevance of the findings.

The obtained results, due to the exploratory character of the analysis, also define several important directions for future research. Prospective studies on larger, multicenter cohorts with balanced representation of tumor subsites are needed to validate the prognostic significance of the analyzed immune markers. In addition, future investigations should incorporate standardized digital pathology or spatially resolved techniques to better capture intratumoral immune heterogeneity. Longitudinal and treatment-stratified analyses may further clarify the dynamic relationship between therapy, HPV status, and immune microenvironment modulation. Ultimately, such approaches may contribute to the development of more precise, biomarker-driven stratification strategies in head and neck cancer.

## 5. Conclusions

In summary, this study, due to its exploratory character, suggests that immune checkpoint markers and tumor-infiltrating lymphocytes are associated with disease-free survival in HNSCC, particularly in HPV16-negative tumors. The absence of PD-L1 overexpression and the presence of CD4 and CD8 overexpression constitute independent favorable prognostic factors, highlighting the pivotal role of the tumor immune microenvironment in disease progression and supporting the further development of immunologically based prognostic models in HNSCC.

## Figures and Tables

**Table 1:** Relation between PD-L1, PD-1, CD3, CD4 or CD8 overexpression and epidemiological, clinical and histopathological features of 155 patients with squamous cell carcinoma of head and neck.

Variables	All *n* (%) ^a^	PD-L1 Overexpression—TPS or CPS			PD-1 Overexpression—CPS			CD3 Overexpression—CPS			CD4 Overexpression—CPS			CD8 Overexpression—CPS		
		Yes *n* (%) ^b^	No *n* (%)	*p* Level ^c^	Yes *n* (%)	No *n* (%)	*p* Level ^c^	Yes *n* (%)	No *n* (%)	*p* Level ^c^	Yes *n* (%)	No *n* (%)	*p* Level ^c^	Yes *n* (%)	No *n* (%)	*p* Level ^c^
All	155 (100.0)	30 (19.4)	125 (80.6)		99 (63.9)	56 (36.1)		79 (51.0)	76 (49.0)		32 (20.6)	123 (79.4)		50 (32.3)	105 (67.7)	
Age																
≤59 years ^d^	51 (32.9)	11 (21.6)	40 (78.4)	0.625	35 (68.6)	16 (31.4)	0.388	21 (41.2)	30 (58.8)	0.088	8 (15.7)	43 (84.3)	0.285	10 (19.6)	41 (80.4)	0.018
>59 years	104 (67.1)	19 (18.3)	85 (81.7)		64 (61.5)	40 (38.5)		58 (55.8)	46 (44.2)		24 (23.1)	80 (76.9)		40 (38.5)	64 (61.5)	
Gender																
Male	130 (83.9)	27 (20.8)	103 (79.2)	0.309	84 (64.6)	46 (35.4)	0.660	65 (50.0)	65 (50.0)	0.583	27 (20.8)	103 (79.2)	0.931	36 (27.7)	94 (72.3)	0.005
Female	25 (16.1)	3 (12.0)	22 (88.0)		15 (60.0)	10 (40.0)		14 (56.0)	11 (44.0)		5 (20.0)	20 (80.0)		14 (56.0)	11 (44.0)	
Status in the Karnofsky scale																
≤80% ^c^	90 (58.1)	16 (17.8)	74 (82.2)	0.559	62 (68.9)	28 (31.1)	0.126	45 (50.0)	45 (50.0)	0.776	24 (26.7)	66 (73.3)	0.029	27 (30.0)	63 (70.0)	0.479
>80%	65 (41.9)	14 (21.5)	51 (78.5)		37 (56.9)	28 (43.1)		34 (52.3)	31 (47.7)		8 (12.3)	57 (87.7)		23 (35.4)	42 (64.6)	
Localization																
Oral cavity	25 (16.1)	5 (20.0)	20 (80.0)	0.393	13 (52.0)	12 (48.0)	0.436	11 (44.0)	14 (56.0)	0.585	6 (24.0)	19 (76.0)	0.000	9 (36.0)	16 (64.0)	0.057
Oropharynx	66 (42.6)	9 (16.6)	57 (86.4)		43 (65.2)	23 (34.8)		37 (56.1)	29 (43.9)		4 (6.1)	62 (93.9)		28 (42.4)	38 (57.6)	
Hypopharynx	6 (3.9)	1 (16.7)	5 (83.3)		3 (50.0)	3 (50.0		2 (33.3)	4 (66.7)		0 (0.0)	6 (100.0)		1 (16.7)	5 (83.3)	
Larynx	58 (37.4)	15 (25.9)	43 (74.1)		40 (69.0)	18 (31.0)		29 (50.0)	29 (50.0)		22 (37.9)	36 (62.1)		12 (20.7)	46 (79.3)	
The level of smoking-Brinkman index ^d^																
≤520 ^e^	33 (21.3)	2 (6.1)	31 (93.4)	0.029	25 (75.8)	8 (24.2)	0.109	16 (48.5)	17 (51.5)	0.748	5 (15.2)	28 (84.8)	0.379	12 (36.4)	21 (63.6)	0.569
>520	122 (78.7)	28 (23.0)	94 (77.0)		74 (60.7)	48 (39.3)		63 (51.6)	59 (48.4)		27 (22.1)	95 (77.9)		38 (31.2)	84 (68.8)	
The level of drinking ^f^																
Low	67 (43.2)	14 (20.9)	53 (79.1)	0.672	42 (62.7)	25 (37.3)	0.789	37 (55.2)	30 (44.8)	0.355	13 (19.4)	54 (80.6)	0.739	26 (38.8)	41 (61.2)	0.128
High	88 (56.8)	16 (18.2)	72 (81.8)		57 (64.8)	31 (35.2)		42 (47.7)	46 (52.3)		19 (21.6)	69 (78.4)		24 (27.3)	64 (72.7)	
T stage																
1	2 (1.3)	0.0 (0.0)	2 (100.0)	0.374	1 (50.0)	1 (50.0)	0.918	1 (50.0)	1 (50.0)	0.465	0 (0.0)	2 (100.0)	0.367	0 (0.0)	2 (100.0)	0.034
2	27 (17.4)	5 (18.5)	22 (81.5)		16 (59.3)	11 (40.7)		17 (63.0)	10 (37.0)		3 (11.1)	24 (88.9)		13 (48.1)	14 (51.9)	
3	78 (50.3)	12 (15.4)	66 (84.6)		51 (65.4)	27 (34.6)		40 (51.3)	38 (48.7)		16 (20.5)	62 (79.5)		28 (35.9)	50 (64.1)	
4	48 (31.0)	13 (27.1)	35 (72.9)		31 (64.6)	17 (35.4)		21 (43.7)	27 (56.3)		13 (27.1)	35 (72.9)		9 (18.7)	39 (81.3)	
N stage																
0	32 (20.6)	7 (21.9)	25 (78.1)	0.426	22 (68.8)	10 (31.2)	0.227	16 (50.0)	16 (50.0)	0.678	8 (25.0)	24 (75.0)	0.769	12 (37.5)	20 (62.5)	0.705
1	28 (18.1)	8 (28.6)	20 (71.4)		17 (60.7)	11 (39.3)		12 (42.9)	16 (57.1)		7 (25.0)	21 (75.0)		9 (32.1)	19 (67.9)	
2	83 (54.2)	14 (16.7)	70 (83.3)		56 (66.7)	28 (33.3)		44 (52.4)	40 (47.6)		15 (17.9)	69 (82.1)		27 (32.1)	57 (67.9)	
3	11 (7.1)	1 (9.1)	10 (90.9)		4 (36.4)	7 (63.6)		7 (63.6)	4 (36.4)		2 (18.2)	9 (81.8)		2 (18.2)	9 (81.8)	
Grade																
1	48 (31.0)	7 (16.6)	41 (85.4)	0.021	28 (58.3)	20 (41.7)	0.273	28 (58.3)	20 (41.7)	0.365	8 (16.7)	40 (83.3)	0.138	16 (33.3)	32 (66.7)	0.122
2	85 (54.8)	14 (16.5)	71 (83.5)		59 (69.4)	26 (30.6)		42 (49.4)	43 (50.6)		16 (18.8)	69 (81.2)		31 (36.5)	54 (63.5)	
3	22 (14.2)	9 (40.9)	13 (59.1.)		12 (54.6)	10 (45.4)		9 (40.9)	13 (59.1)		8 (36.4)	14 (63.6)		3 (13.6)	19 (86.4)	
Keratinization																
Yes	44 (57.1)	32 (72.7)	12 (27.3)	0.505	39 (60.9)	25 (39.1)	0.524	37 (57.8)	27 (42.2)	0.153	19 (20.9)	72 (79.1)	0.932	29 (31.9)	62 (68.1)	0.901
No	33 (42.9)	23 (69.7)	10 (30.3)		60 (65.9)	31 (34.1)		42 (46.1)	49 (53.8)		13 (20.3)	51 (79.7)		21 (32.8)	43 (67.2)	
HPV16 active infection																
Yes	28 (18.5)	6 (21.4)	22 (78.6)	0.741	19 (67.9)	9 (32.1)	0.658	19 (67.9)	9 (32.1)	0.040	2 (7.1)	26 (92.9)	0.044	14 (50.0)	14 (50.0)	0.017
No	123 (81.5)	23 (18.7)	100 (81.3)		78 (63.4)	45 (36.6)		57 (46.3)	66 (53.7)		30 (24.4)	93 (75.6)		33 (26.8)	90 (73.2)	
PD-L1 overexpression																
Yes	30 (19.4)	-	-	-	7 (23.3)	23 (76.7)	0.000	10 (33.3)	20 (66.7)	0.031	6 (20.0)	24 (80.0)	0.923	9 (30.0)	21 (70.0)	0.768
No	125 (80.6)	-	-		92 (73.6)	33 (26.4)		69 (55.2)	56 (44.8)		26 (20.8)	99 (79.2)		41 (32.8)	84 (67.2)	
PD-1 overexpression																
Yes	99 (63.9)	7 (7.1)	92 (92.9)	0.000	-	-	-	51 (51.5)	48 (48.5)	0.856	23 (23.2)	76 (76.8)	0.290	37 (37.4)	62 (62.6)	0.070
No	56 (36.1)	23 (41.1)	33 (58.9)		-	-		28 (50.0)	28 (50.0)		9 (16.1)	47 (83.9)		13 (23.1)	43 (76.8)	
CD3 overexpression																
Yes	79 (51.0)	10 (12.7)	69 (87.3)	0.031	51 (64.6)	28 (35.4)	0.856	-	-	-	20 (25.3)	59 (74.7)	0.143	33 (41.8)	46 (58.2)	0.010
No	76 (49.0)	20 (26.3)	56 (73.7)		48 (63.2)	28 (36.8)		-	-		12 (15.8)	64 (84.2)		17 (22.4)	59 (77.6)	
CD4 overexpression																
Yes	32 (20.6)	6 (18.7)	26 (81.3)	0.923	23 (71.9)	9 (28.1)	0.290	20 (62.5)	12 (37.5)	0.143	-	-	-	11 (34.4)	21 (65.6)	0.774
No	123 (79.3)	24 (19.5)	99 (80.5)		76 (76.8)	47 (38.2)		59 (48.0)	64 (52.0)		-	-		39 (31.7)	84 (68.3)	
CD8 overexpression																
Yes	50 (32.3)	9 (18.0)	41 (82.0)	0.768	37 (74.0)	13 (26.0)	0.070	33 (66.0)	17 (34.0)	0.010	11 (22.0)	39 (78.0)	0.774	-	-	-
No	105 (67.4)	21 (20.0)	84 (80.0)		62 (59.1)	43 (40.9)		46 (43.8)	59 (56.2)		21 (20.0)	84 (80.0)		-	-	
Treatment																
Definitive CisPt-CRT or surgery + CisPt-CRT	45 (29.0)	8 (18.8)	37 (82.2)	0.665	27 (60.0)	18 (40.0)	0.798	30 (66.7)	15 (33.3)	0.059	3 (6.7)	42 (93.3)	0.052	17 (37.8)	28 (62.2)	0.613
Definitive RT or surgery + RT	88 (56.8)	19 (21.6)	69 (78.4)		58 (65.9)	30 (34.1)		40 (45.4)	48 (54.6)		27 (30.7)	61 (69.3)		27 (30.7)	61 (69.3)	
Induction CT + definitive RT	22 (14.2)	3 (13.6)	19 (86.4)		14 (63.6)	8 (36.4))		9 (40.9)	13 (59.1)		2 (9.1)	20 (90.9)		6 (27.3)	16 (72.7)	
Treatment outcome																
Regression of cancer disease	61 (39.4)	7 (11.5)	54 (88.5)	0.019	44 (72.1)	17 (27.9)	0.006	43 (70.5)	18 (29.5)	0.000	14 (22.9)	47 (77.1)	0.001	31 (50.8)	30 (49.2)	0.000
Treatment failure	34 (21.9)	11 (32.4)	8 (67.4)		23 (67.6)	11 (32.4)		9 (26.5)	25 (73.5)		0 (0.0)	34 (100.0)		0 (0.0)	34 (100.0)	
Local recurrence	16 (10.3)	5 (31.3)	11 (68.7)		6 (37.5)	10 (62.5)		3 (18.7)	13 (81.3)		3 (18.7)	13 (81.3)		2 (12.5)	14 (87.5)	
Distant metastases	7 (4.5)	3 (42.9)	4 (57.1)		1 (14.3)	6 (85.7)		1 (14.3)	6 (85.7)		0 (0.0)	7 (100.0)		0 (0.0)	7 (100.0)	
Non-cancer reasons of death	37 (23.9)	4 (10.8)	33 (89.2)		25 (67.6)	12 (32.4)					15 (40.5)	22 (59.5)		17 (45.9)	20 (54.1)	
Survival																
Alive at the last follow-up	68 (43.9)	9 (13.2)	59 (86.8)		48 (70.6)	20 (29.4)	0.100	45 (66.2)	23 (33.8)	0.000	15 (22.1)	53 (77.9)	0.000	31 (45.6)	37 (54.4)	0.000
Death from cancer disease	50 (32.3)	17 (34.0)	33 (66.0)		26 (52.0)	24 (48.0)		11 (22.0)	39 (78.0)		2 (4.0)	48 (96.0)		2 (4.0)	48 (96.0)	
Death from others reasons	37 (23.8)	4 (10.8)	33 (89.2)		25 (67.6)	12 (32.4)		23 (62.2)	14 (37.8)		15 (40.5)	33 (59.5)		17 (46.0)	20 (54.0)	

^a^ Column percentage, ^b^ Row percentage, ^c^ Pearson χ^2^ test, ^d^ Number of cigarettes per day × years of smoking, ^e^ Median values, ^f^ Low level of drinking—no alcohol and occasional drinkers (at most two drinks a day, especially with a meal), high level of drinking—more than 15 drinks of high percentage alcohol in a week and alcoholics; CisPt-CRT: concurrent chemoradiotherapy with cisplatin; CT: chemotherapy.

**Table 2:** Univariate Cox proportional hazard model for 5-year disease-free survival of 155 patients with squamous cell carcinoma of oral cavity and oropharynx and in the subgroup of HPV16-positive and HPV16-negative patients.

Variables	Whole Group of Patients		HPV16 (+)		HPV16 (−)	
	Without Progression/ Progression *n* (%)	Log-Rank *p*	Without Progression/ Progression *n* (%)	Log-Rank *p*	Without Progression/ Progression *n* (%)	Log-Rank *p*
Age						
≤59 years ^a^	27/24 (52.9)	0.028	21/2 (91.3)	0.505	21/24 (46.7)	0.076
>59 years	76/28 (73.1)		5/0 (100.0)		52/26 (66.7)	
Gender						
Male	82/48 (63.1)	0.019	17/2 (89.5)	0.367	63/46 (57.8)	0.125
Female	21/4 (84.0)		9/0 (100.0)		10/4 (71.4)	
Status in the Karnofsky scale						
≤80% ^c^	58/32 (64.4)	0.384	9/2 (81.8)	0.071	48/30 (61.5)	0.671
>80%	45/20 (69.2)		17/0 (100.0)		25/20 (55.6)	
Localization						
Oral cavity + Oropharynx	64/28 (69.6/30.4)	0.415	25/2 (92.6/7.4)	0.756	35/26 (57.4/42.6)	0.487
Hypopharynx + Larynx	39/24 (61.9/38.1)		1/0 (100.0/0.0)		28/24 (61.3/38.7)	
The level of smoking—Brinkman index ^b^						
≤520 ^c^	28/5 (84.9/15.1) 75/47 61.5/38.5)	0.012	14/0 (100.0)	0.156	12/5 (70.6)	0.292
>520			12/2 (85.7)		61/45 (57.5)	
The level of drinking ^c^						
Low	50/17 (74.6/25.4)	0.043	20/0 (100.0/0)	0.406	27/17 (61.4/38.6)	0.555
High	53/35 (60.2/39.8)		6/2 (75.0/25.0)		46/33 (58.2/41.8)	
T stage						
1 + 2	25/4 (86.2/13.8)	0.006	8/0 (100.0/0.0)	0.381	16/4 (80.0/20.0)	0.017
3 + 4	78/48 (61.9/38.1)		18/2 (90.0/10.0)		57/46 (55.3/44.7)	
N stage						
0	23/9 (71.9/28.1)	0.243	2/0 (100.0/0.0)	0.655	21/9 (70.0/30.0)	0.060
1 + 2 + 3	80/43 (66.0/35.0)		24/2 (92.3/7.7)		52/41 (55.9/44.1)	
Grade						
1	33/15 (68.7/31.3)	0.457	9/1 (90.0/10.0)	0.631	24/14 (63.2/36.8)	0.355
2 + 3	58/27 (68.2/31.8)		17/1 (94.4/5.6)		49/36 (57.6/42.4)	
Keratinization						
Yes	46/18 (71.9/28.1)	0.184	16/1 (94.1/5.9)	0.724	26/17 (60.5/39.5)	0.798
No	57/34 (62.6/37.4)		10/1 (90.9/9.1)		47/33 (58.8/41.2	
HPV16 infection						
Yes	26/2 (92.9/7.1)	0.001	-	-	-	-
No	73/50 (59.3/40.6)		-		-	
PD-L1 overexpression						
Yes	14/16 (46.7/53.3)	0.008	6/0 (100.0)	0.446	7/16 (30.4/69.6)	0.001
No	89/36 (71.2/28.8)		20/2 (90.9)		66/34 (66.0/34.0)	
PD-1 overexpression						
Yes	71/28 (71.7/28.3	0.031	18/1 (94.7/5.3)	0.538	51/27 (65.4/34.6)	0.030
No	32/24 (57.1/42.9)		8/1 (88.9/11.1)		22/23 (48.9/51.1)	
CD3 overexpression						
Yes	66/12 (84.6/15.4)	0.000	18/1 (94.7/5.3)	0.538	45/11 (80.4/19.6)	0.000
No	36/40 (47.4/52.6)		8/1 (88.9/11.1)		27/39 (40.9/59.1)	
CD4 overexpression						
Yes	30/2 (93.7/6.3)	0.001	24/2 (93.3/7.7)	0.748	28/2 (93.3/6/7)	0.000
No	73/50 (59.3/40.7)		2/0 (100.0/0.0)		45/48 (48.4/51.6)	
CD8 overexpression						
Yes	48/2 (96.0/4.0)	0.000	14/0 (100.0/0.0)	0.176	31/2 (93.9/6.1)	0.000
No	55/50 (52.4/47.6)		12/2 (85.7/14.3)		42/48 (46.7/53.3)	
Treatment						
Definitive CisPt-CRT or surgery + CisPt-CRT	36/9 (80.0/20.0)	0.012	16/0 (100.0/0.0)	0.136	17/9 (65.4/34.6)	0.114
Definitive RT or surgery + RT	57/31 (64.8/35.2)		7/1 (87.5/12.5)		50/30 (62.5/37.5)	
Induction CT + definitive RT	10/12 (45.5/54.5)		3/1 (75.0/25.0)		6/11 (35.3/64.7)	

^a^ Median values, ^b^ Number of cigarettes per day × years of smoking. ^c^ Low level of drinking—no alcohol and occasional drinkers (at most two drinks a day, especially with a meal), high level of drinking—more than 15 drinks of high percentage alcohol in a week and alcoholics. CisPt-CRT: concurrent chemoradiotherapy with cisplatin; CT: chemotherapy.

**Table 3:** Multivariate Cox proportional hazard model for disease-free survival of 155 patients with squamous cell carcinoma of head and neck cancers and in the subgroup of HPV16-negative patients.

	Whole Group			HPV16 (−)		
	HR	95% CI	Log-Rank *p* ^a^	HR	95% CI	Log-Rank *p* ^a^
HPV16 infection						
Yes	1.000	0.030–0.574	0.007			-
No						
PD-L1 overexpression						
Yes	1.340	2.032–7.175	0.000	1.341	2.054–7.110	0.001
No	1.000			1.000		
CD4 overexpression						
Yes	1.000	0.015–0.276	0.000	1.000	0.018–0.324	0.000
No						
CD8 overexpression						
Yes	1.000		0.000	1.000	0.022–0.381	0.000
No						
Treatment						
Definitive CisPt-CRT or surgery + CisPt-CRT	1.000	1.113–4.849	0.025			
Definitive RT or surgery + RT or induction CT + definitive RT	1.1862					

^a^ *p*-values were examined by the Cox proportional hazard model for multivariate survival analysis. HR, hazard ratio; CI, confidence interval.

## Data Availability

The data are available from the corresponding author upon reasonable request.
